# Species-Dependent Splice Recognition of a Cryptic Exon Resulting from a Recurrent Intronic *CEP290* Mutation that Causes Congenital Blindness

**DOI:** 10.3390/ijms16035285

**Published:** 2015-03-09

**Authors:** Alejandro Garanto, Lonneke Duijkers, Rob W. J. Collin

**Affiliations:** 1 Department of Human Genetics, Radboud University Medical Center, Geert Grooteplein Zuid 10, 6525 GA Nijmegen, The Netherlands; E-Mails: alex.garantoiglesias@radboudumc.nl (A.G.); lonneke.duijkers@radboudumc.nl (L.D.); 2 Radboud Institute for Molecular Life Sciences, Radboud University Medical Center, 6525 GA Nijmegen, The Netherlands

**Keywords:** *CEP290*, deep-intronic mutation, pre-mRNA splicing, Leber congenital amaurosis

## Abstract

A mutation in intron 26 of *CEP290* (c.2991+1655A>G) is the most common genetic cause of Leber congenital amaurosis (LCA), a severe type of inherited retinal degeneration. This mutation creates a cryptic splice donor site, resulting in the insertion of an aberrant exon (exon X) into ~50% of all *CEP290* transcripts. A humanized mouse model with this mutation did not recapitulate the aberrant *CEP290* splicing observed in LCA patients, suggesting differential recognition of cryptic splice sites between species. To further assess this phenomenon, we generated two *CEP290* minigene constructs, with and without the intronic mutation, and transfected these in cell lines of various species. RT-PCR analysis revealed that exon X is well recognized by the splicing machinery in human and non-human primate cell lines. Intriguingly, this recognition decreases in cell lines derived from species such as dog and rodents, and it is completely absent in *Drosophila*. In addition, other cryptic splicing events corresponding to sequences in intron 26 of *CEP290* were observed to varying degrees in the different cell lines. Together, these results highlight the complexity of splice site recognition among different species, and show that care is warranted when generating animal models to mimic splice site mutations *in vivo*.

## 1. Introduction

*CEP290* encodes the CEntrosomal Protein of 290 kDa, a protein that is thought to play an important role in ciliogenesis and/or ciliary transport, in many different cell types including retinal photoreceptor cells [1,2,3]. Mutations in *CEP290* gene have been associated with a wide range of ciliopathies, ranging from lethal syndromes (Meckel-Grüber syndrome MIM#611134) to non-syndromic retinal degeneration (Leber congenital amaurosis MIM#611755) [4,5,6,7]. Leber congenital amaurosis (LCA) is an early-onset severe form of visual impairment that can be caused by mutations in either one of at least 22 different genes (RetNet: https://sph.uth.edu/retnet). Interestingly, mutations in *CEP290* underlie approximately 20% of the cases, with a recurrent intronic mutation (c.2991+1655A>G) accounting for up to 15% of all LCA cases in some European and North-American populations [5,8]. This deep-intronic *CEP290* mutation creates a splice donor site that allows the insertion of a 128-bp cryptic exon to approximately 50% of the *CEP290* transcripts, resulting in premature termination of protein synthesis [5,9].

The generation of a mature mRNA molecule involves multiple steps. First, DNA is transcribed to pre-mRNA, and subsequently the splicing machinery carefully removes the introns to produce a mature mRNA that will be translated by the ribosomes [10,11]. Several signal sequences throughout the pre-mRNA direct the binding of proteins of the spliceosome [10,12]. Mainly, exon-intron boundaries are delimited by consensus splice donor and acceptor sequences. However, besides these canonical sequences, other signals such as enhancers or repressors, as well as non-canonical signals can lead to the excision or inclusion of an exon, leading to what is called alternative splicing. This process increases the complexity of gene expression and allows the generation of multiple protein products derived from the same gene [11,12,13]. In addition, the secondary structure of the pre-mRNA can play an important role in the accessibility of splice factors and thereby regulate splicing [14].

Besides naturally occurring alternative splicing, genetic mutations can also alter the composition of mRNA molecules. Several mutations in the exon-intron boundaries that result in exon skipping or intron retention are known to underlie a plethora of different inherited conditions, including retinal degeneration [15]. In addition, deep-intronic mutations may activate cryptic splice acceptor or donor sites, resulting in the insertion of so-called pseudo-exons to the final mRNA transcript, often leading to premature termination of the corresponding protein [15]. One of the most recurrent examples of these is the aforementioned deep-intronic mutation in *CEP290* (c.2991+1655A>G) that generates a splice donor site resulting in the insertion of a cryptic exon (coined exon X) into ~50% of the *CEP290* mRNA transcripts [5].

Recently, we generated a humanized mouse model carrying this intronic mutation in order to mimic the molecular and phenotypic characteristics of *CEP290*-associated LCA. Unfortunately, this model did not recapitulate the aberrant *CEP290* splicing that we observe in LCA patients with this mutation [16]. On one hand, exon X was inserted into only a small proportion of *Cep290* transcripts in the retina of the transgenic mice, whereas in addition, a second cryptic exon (exon Y) within the human intron 26 of *CEP290* was spliced into part of the *Cep290* transcripts. The total amount of aberrant *Cep290* transcripts (containing either exon X, exon Y or exons X + Y) did not exceed ~15% of the total pool of *Cep290* transcripts, and therefore did not result in any signs of retinal degeneration in our mouse model [16]. Together, these data suggested a differential recognition of cryptic splice sites between species. Here, we further studied this phenomenon, and show that the recognition of the cryptic exon introduced by the c.2991+1655A>G mutation in *CEP290* indeed is species-dependent, and seems to correlate to the evolutionary distance to humans. In addition, we show that strengthening the splice acceptor and donor sites of exon X by site-directed mutagenesis allows an efficient recognition of exon X in murine cells, highlighting the differences between the human and murine splicing machineries, and thereby providing important insights in how to study human phenotypes caused by splice site mutations.

## 2. Results

### 2.1. Generation and Validation of CEP290 Minigenes

**Figure 1:**
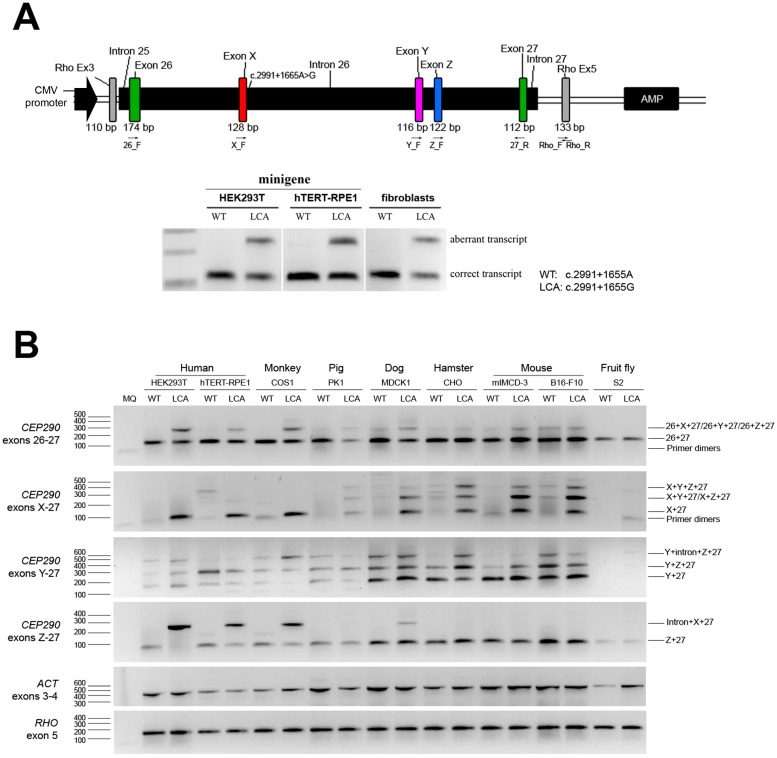
Assessment of *CEP290* splicing upon overexpression of the minigenes in cells from different species. (**A**) **Upper** panel: Schematic representation of the position of the promoter and different cryptic exons within intron 26, as well as the primer localization in the wild-type (WT) and Leber congenital amaurosis (LCA), minigene construct carrying the intronic mutation; **Lower** panel: Splicing pattern detected after minigene expression in human HEK293T and hTERT-RPE1 cells compared to fibroblast cell lines of a healthy individual or an individual homozygously carrying the intronic *CEP290* mutation; (**B**) Assessment of the splicing pattern of WT and LCA minigenes in several cell lines of different species via RT-PCR analysis. Actin (*ACT*) and rhodopsin (*RHO*) were used to normalize samples. MQ represents milli-Q water and was the negative control of the PCR reaction. For semiquantitative analysis of the gels, see Supplementary Figure S1.

In order to evaluate whether the recognition of the cryptic splice donor site that is activated by the c.2991+1655A>G mutation in *CEP290* indeed is species-dependent, two *CEP290* minigenes encompassing the genomic region between exons 26 and 27 of *CEP290* under the control of the cytomegalovirus (CMV) immediate-early promoter were generated; one of them was carrying the c.2991+1655A>G mutation (Figure 1A). To assess whether transfection of these minigene constructs recapitulates the splice pattern observed in individuals with *CEP290*-associated LCA, human embryonic kidney cells (HEK293T) and human retinal pigmented epithelium cells (hTERT-RPE1) were transfected. RT-PCR analysis revealed that transfection of the LCA *CEP290* minigene resulted in the exact same transcript composition as the one observed in patient-derived fibroblast cells, demonstrating the suitability of this cellular system to assess *CEP290* pre-mRNA splicing (Figure 1A, Supplementary Figure S1A).

### 2.2. Assessment of Cryptic Splice Events in Different Species

In the humanized LCA mouse model *Cep290^lca/lca^*, exon X was only poorly recognized by the murine splicing machinery. In addition, a new aberrant exon (exon Y) residing in intron 26 of *CEP290* (Figure 1A) was included in a small proportion of the *CEP290* transcripts, both in the *Cep290^lca/lca^* as well as in the humanized control *Cep290^hum/hum^* model [16]. To study the recognition of the splice site sequences that define these aberrant exons by the splicing machinery of different organisms, cell lines from various species (listed in Table 1) were transfected with either the WT or the LCA minigene construct. A series of dedicated RT-PCR analysis (with primers located in exon 26, exon X, exon Y and exon 27, Figure 1B) was performed, followed by Sanger sequencing analysis of all the PCR products that were identified on gel, and semiquantitative analysis of all the bands. PCR analysis with primers 26_F and 27_R revealed that in all species, the majority of *CEP290* transcripts represented the wild-type mRNA with a minimal insertion of cryptic exons. Only in the human and monkey-derived cell lines transfected with the LCA minigene, a robust insertion of a cryptic exon was observed, therefore most likely representing exon X (Figure 1B, upper panel and Supplementary Figure S1B, upper graph). In a more dedicated analysis using primers located inside the cryptic exons, transcripts containing exon X were detected in primate-derived cell lines but were also identified in cell lines from pig, dog, hamster and mouse but not the fruit fly. In contrast, exon Y-containing transcripts were only barely detectable in human, primate and porcine cell lines, whereas canine and rodent cells showed a high recognition of exon Y. In *Drosophila*, this cryptic exon Y was also not recognized (Figure 1B and Supplementary Figure S1B). Following Sanger sequencing analysis of the PCR products, a third cryptic exon, coined exon Z, was identified, located downstream of exon Y (Figure 1A). This exon was recognized by the splicing machinery in cell lines of all species, but mostly in the dog and rodent cells, without major differences between WT and LCA constructs (Figure 1B and Supplementary Figure S1B). The occurrence of exon Z was subsequently also tested in fibroblast cells derived from a patient with the intronic *CEP290* mutation but no exon Z-containing transcripts were amplified. In the retinas of the humanized mouse models (*Cep290^lca/lca^* and *Cep290^hum/hum^*) however, trace amounts of exon Z-containing transcripts were found (data not shown), indicating that the inclusion of exon Z, albeit in very low levels, is not an artifact solely caused by the overexpression of the minigene. In the PCR analysis using primers Z_F and 27_R, a ~300-bp PCR product was identified in some samples. Sequence analysis revealed that this product represented a part of intron 26 (just upstream of exon X, the sequence of exon X and part of the sequence of exon 27). Due to the high degree of sequence identity between the primer-binding region of exon Z, and another region in intron 26, in those cell lines that express a high amount of exon X-containing *CEP290* transcripts (*i.e.*, human, monkey), apparently this product is efficiently amplified. None of these intron-retaining transcripts however were found in the patient-derived cells or in the retinas of the humanized mouse models (data not shown), suggesting that these products are the result of the overexpression of the minigene constructs and therefore have no physiological relevance.

### 2.3. Identification of a Suitable Sequence to Increase the Recognition of Exon X in Mouse

**Figure 2:**
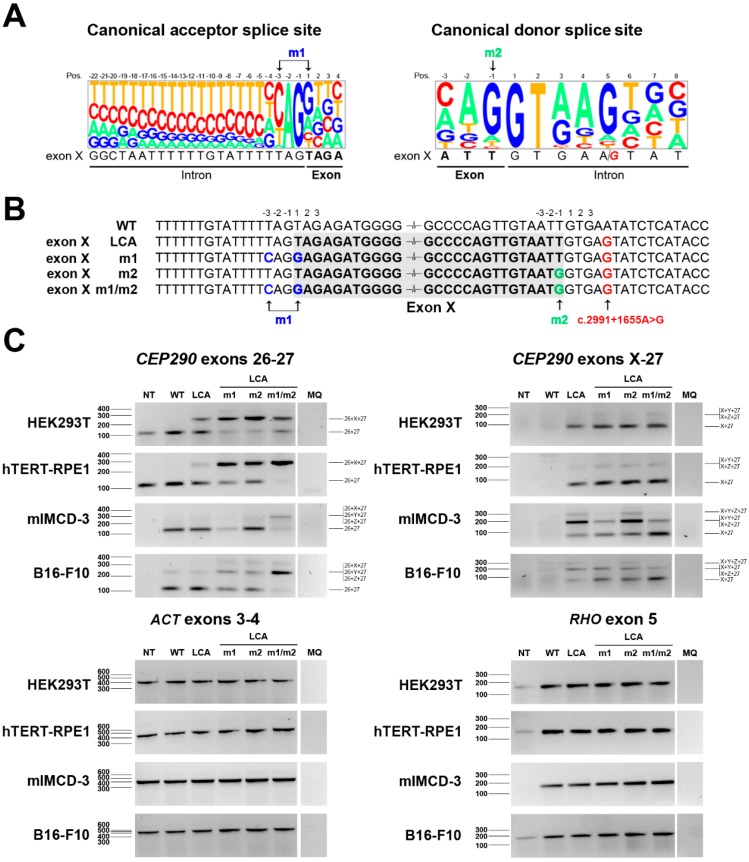
Evaluation of the modification of the splice sites of exon X. (**A**) Sequence alignment of the splice site sequences of exon X with the consensus canonical acceptor and donor splice sites; (**B**) Representation of the sequences surrounding exon X and the mutations introduces (m1: blue; m2: green; c.2991+1655A>G in red) in all the constructs generated; (**C**) Evaluation of the splicing pattern observed in these constructs after overexpression in two human (HEK293T and hTERT-RPE1) and two murine (mIMCD-3 and B16-F10) cell lines. Amplification of actin (*ACT*) and rhodopsin (*RHO*) was used for normalization and to assess transfection efficiency, respectively. Milli-Q water (MQ) was used as negative control of the PCR. Semiquantitative analysis of the gels is shown in Supplementary Figure S2.

Having studied the species-dependent recognition of cryptic *CEP290* exons in detail, we next aimed to assess whether further modification of important splice site sequences would increase the recognition of cryptic exon X in mouse cells. A comparison of the acceptor and donor splice site sequences of exon X to the mammalian consensus sequence highlighted a few differences (Figure 2A). In order to make the acceptor and donor splice sites of exon X more similar to the consensus sequence, we performed site-directed mutagenesis, generating three additional minigene constructs. The first one (LCA-m1) included two changes in the splice acceptor site (at position −3T>C and +1T>G), in a second one (LCA-m2) the last nucleotide of the exon was changed (−1T>G), and the final construct combined these changes (LCA-m1/m2) (Figure 2B).

**Table 1:** Oligonucleotide sequences.

Name	Forward Primers (Sequence 5'–3')
*Act*_F	ACTGGGACGACATGGAGAAG
*Rho*_F	ATCTGCTGCGGCAAGAAC
*CEP290*_attB1_i25_F	GGGGACAAGTTTGTACAAAAAAGCAGGCTTCGGCCGCTCTTTCTCAAAAGTGGC
*CEP290*_m1_F	GCCCGGCTAATTTTTTGTATTTTCAGGAGAGATGGGGTTTCACCTTG
*CEP290*_m2_F	CACCTGGCCCCAGTTGTAATGGTGAGTATCTCATACCTATCCC
*CEP290*__F	TGCTAAGTACAGGGACATCTTGC
*CEP290*_X_F	GCACCTGGCCCCAGTTG
*CEP290*_Y_F	CATAGCTCATTGCAGCCTTG
*CEP290*_Z_F	TGCCTCAGTCTCCTGAGTAG
	**Reverse Primers (Sequence 5'–3')**
*Act*_R	TCTCAGCTGTGGTGGTGAAG
*Rho*_R	AGGTGTAGGGGATGGGAGAC
*CEP290*_attB2_i27_R	GGGGACCACTTTGTACAAGAAAGCTGGGTGCTTGGTGGGGTTAAGTACAGG
*CEP290*_m1_R	CAAGGTGAAACCCCATCTCTCCTGAAAATACAAAAAATTAGCCGGGC
*CEP290*_m2_R	GGGATAGGTATGAGATACTCACCATTACAACTGGGGCCAGGTG
*CEP290*_27_R	AGACTCCACTTGTTCTTTTAAGGAG

Following site-directed mutagenesis, the five different minigene constructs (WT, LCA, LCA-m1, LCA-m2 and LCA-m1/m2) were transfected in two cell lines of human (HEK293 and hTERT-RPE1) and two cell lines of mouse (mIMCD-3 and BL16-F10) origin. RT-PCR analysis using primers located in exon 26 and exon 27 revealed that in the human cell lines, strengthening either the acceptor or the donor site of exon X already resulted in a clear increase of aberrant transcripts (Figure 2C and Supplementary Figure S2A,B). Similar effects were also observed in monkey COS1 cells (data not shown). In contrast, in the mouse cell lines, an obvious increase in aberrant transcripts is only observed if both splice sites are modified, whereas only a minor shift was observed if only one of the two was mutated (Figure 2C and Supplementary Figure S2A,B). When the primer was specifically located in exon X, the previous results were supported by an increasing amount of exon X detected in the human cell lines. Interestingly, in the mouse cell lines, the levels of exon X-containing transcripts without any other cryptic exons (*i.e.*, exons Y and/or Z) only increased if both the splice acceptor (m1) as well as the splice donor (m2) site of exon X are mutated (Figure 2C and Supplementary Figure S2B), supporting the fact that the increased aberrant transcripts detected in PCR from exon 26 to exon 27 included the cryptic exon X. These data again highlight the differences in splice site recognition between humans and mice, but also show that by site-directed mutagenesis of splice site sequences, it is possible to modulate the recognition of cryptic exons in different species.

## 3. Discussion

In this study, we have evaluated the recognition of cryptic splice sites and subsequent insertion of cryptic exons to *CEP290* mRNA in a series of cell lines from different species, in order to better understand the splicing pattern observed in the humanized *CEP290* mouse models [16]. Besides a clear differential recognition of a disease-associated pseudo-exon X between different species, we have shown that modifying splice-site sequences allows shifting the balance of multiple splice products, providing tools to better mimic the molecular and phenotypic characteristics of splice site mutations.

Differences in splicing patterns among species due to tissue-specific and/or differential alternative splicing have been described in a few occasions [16,17,18,19]. Moreover, the retina is a tissue that displays a high degree of transcriptional activity and alternative splicing, without a full understanding of this transcriptional complexity [17,20,21,22,23]. The degree of alternative splicing appears to be directly correlated with the complexity of the organism, especially for those genes involved in neuronal and immunological development and/or processes [24,25]. Thus, the specificity of the splicing machinery and the signals involved vary not only among lineages, but also within mammals [24,26].

Using minigene constructs in a cellular system, we showed that we could mimic the aberrant *CEP290* splicing that is observed in patient-derived cells [9]. Subsequent transfection of these minigene constructs in different murine cell lines consistently revealed a similar splicing behavior to the one described previously in our *Cep290^hum/hum^* and *Cep290^lca/lca^* mouse models [16]. To determine whether this recognition was exclusively occurring in humans, several cell lines derived from other species were assessed. The fact that the transcript containing exons 26 + X + 27 (the aberrant transcript robustly detected in LCA patients) was also well recognized in primate cells but not efficiently in other cell lines, suggests that either the primate spliceosome is more flexible in recognizing sequences differing from the consensus, or that other signals present within intron 26 as well as other molecular factors exclusively present in these species facilitate the recruiting of the spliceosome and thereby the insertion of exon X in the final *CEP290* transcript. For instance, branch-site sequences can provide some plasticity in determining constitutive or alternative splicing, and it has been shown that these are low conserved between mouse and human [27]. In this context, although exon X-containing transcript levels appear to be similar in all the cell lines, these differences may explain why the aberrant transcript found in LCA patients is poorly recognized in non-primate cell lines. Interestingly, exon X appears to be mainly inserted together with exon Y, suggesting that a more complex co-recognition of splice signals determines the final transcript composition. Intriguingly, with exon Y occurs the opposite effect, it is well recognized in rodents but not endogenously in human fibroblast cells, neither from LCA patients nor from healthy individuals [16]. In human and primate cell lines however, some exon Y-containing transcripts were detected upon transfection of the minigene constructs, which may be explained by the overexpression of the construct. The same applies for exon Z; although it was not possible to detect this exon in fibroblast cells from human individuals, it was present in the retinas of the humanized mouse models, indicating that exon Z it is not solely an artifact of the overexpression. Together, the cellular system we used reflects the *in vivo* situation (Figure 1A), and although the overexpression of the constructs may result in some artificial splicing events, clear differences between species were observed.

The use of the constructs under the control of the CMV immediate-early promoter may add some variability in the expression levels of some of the cell lines. For instance, it is known that the CMV promoter is not optimal for fly cell lines, but works well in mammalian cell lines. In order to facilitate the comparison of all cell lines, we used the CMV promoter also in fly cells, which resulted in the expression of *CEP290* transcripts, as well as rhodospin, indicating that both minigenes were expressed in this cellular model, but perhaps at lower levels than in the other cell lines. In addition, in all non-primate cell lines, the *CEP290* expression detected was derived only from the minigenes, since the primers used are not capable of amplifying endogenous *CEP290* due to several mismatches in the sequence relative to the genome of those species. In primate cell lines, however, endogenous *CEP290* was amplified, explaining ~20%–30% of the total *CEP290* levels in some of the cell lines. This has been taken into account for the semiquantitative analysis.

A “humanization” of animal models has been performed for several disorders [28,29,30,31], however to our knowledge all but ours [16] recapitulated the human splicing pattern, although this did not always lead to a phenotype (*i.e.*, in a *IKBKAP* humanized mouse model) [32]. The fact that our mouse model did not show the aberrant splicing of *CEP290* that is characteristic of the LCA-causing intronic mutation, prevents us to study the exact pathophysiological mechanisms as well as to test pre-clinical efficacy of AON-based splice correction therapy [9,33]. For this reason, we aimed to identify a suitable combination of acceptor and donor splice site sequences that would allow an efficient recognition of cryptic exon X by the mouse splicing machinery. The modification of only one of the splice sites was enough to increase the inclusion of exon X in primate cell lines, but not in mouse, even though the novel splice site sequences were highly similar to the consensus one. In mice, only the combination of both modifications was able to consistently enhance inclusion of the cryptic exon X. These findings could provide us with tools to generate a new humanized mouse model for *CEP290*-associated LCA, suitable to determine pre-clinical proof-of-concept of novel therapeutic strategies *in vivo* [9,33].

Together, our results highlight the complexity of the splicing process among species, and show the importance of taking into account these differences when generating animal models to mimic human disorders caused by splice mutations.

## 4. Experimental Section

### 4.1. Minigene Generation

A PCR with primers located in *CEP290* introns 25 (forward) and 27 (reverse) was performed on genomic DNA of healthy individuals and the PCR product was cloned into a pDONR vector using the Gateway system (primer sequences in Table 1). Via site-directed mutagenesis, the c.2991+1655A>G mutation was introduced. Both pDONR vectors (mutant and wild-type (WT)) were sequenced and cloned into the destination vector pCi-Neo-Rho-Splicing vector, a home-made vector that allows the cloning of the fragment of interest between exons 3 and 5 of *RHO* under the control of the cytomegalovirus immediate-early promoter [34], generating WT or LCA minigene constructs (Figure 1A).

### 4.2. Site-Directed Mutagenesis

Forward and reverse primers were designed to modify specific nucleotides in the acceptor (m1) and donor splice (m2) sites of exon X. Site-directed mutagenesis was performed using the primers listed in Table 1 and the high fidelity Phusion *Taq* polymerase. The PCR program consisted of an initial step of 2 min at 94 °C followed by 15 cycles: 94 °C for 30 s, 50 °C for 30 s and 72 °C for 12 min. Last step was the final extension for 15 min at 72 °C.

### 4.3. Cell Lines

All cell lines and culture conditions are described in Table 2.

### 4.4. Transfection

Cells (150,000–300,000, depending on the size of the cells) were seeded in 12-well plates and transfected the day after with FuGene (Promega, Madison, WI, USA) reagent (ratio 1:3) according to the manufacturer’s protocol. Medium was replaced after 24 h and cells were harvested 48 h post-transfection. Cells were transfected with 1 μg of the WT or LCA minigene for the assessment of the species-dependent recognition, and with WT, LCA or all the modified LCA minigenes for the more in depth study in human and murine cell lines.

### 4.5. RT-PCR and Transcriptional Analysis

RNA isolation was performed using the NucleoSpin RNA kit (Nucleospin RNA II, Macherey-Nagel, Düren, Germany) following manufacturer’s instructions. One microgram of the isolated RNA was used for cDNA synthesis using the iScript cDNA synthesis kit (Bio-Rad, Hercules, CA, USA). Subsequently, cDNA was diluted in H_2_O to a final concentration of 5 ng/μL and used for PCR analysis. All reaction mixtures (25 μL) contained 10 μM of each primer pair (Table 1), 2 μM of dNTPs, 1.5 mM MgCl_2_, 10% Q-solution (Qiagen, Venlo, The Netherlands), 1 U of *Taq* polymerase (Roche, Penzberg, Germany) and 25 ng of diluted cDNA. PCR conditions were 94 °C for 2 min, followed by 35 cycles of 20 s at 94 °C, 30 s at 58 °C and 30 s at 72 °C, with a final extension step of 2 min at 72 °C. *RHO* was amplified as a measure to assess the transfection and transcription efficiency, whereas *ACTN* was amplified to serve as a loading control. All PCR products were resolved on 2% agarose gels. Bands were extracted from the agarose gel and purified using NucleoSpin Gel & PCR Clean-up (Macherey-Nagel). Bands were sent for Sanger sequencing, employing the same primers that were used in the PCR reaction. Semiquantitative analysis of the bands was performed using Image J software [35]. Values were first normalized against actin and subsequently against rhodopsin.

**Table 2:** Cell lines and growth conditions.

Cell Line (Source)	Animal	Tissue of Origin	Culture Medium	Temp.
**Fibroblast** (skin biopsy)	Human	Skin	DMEM supplemented with 20% Fetal Calf Serum (FCS), 1% NaPyr, 100 U/mL penicillin and 100 μg/mL streptomycin	37 °C
**HEK293T** (ATCC^®^ CRL-3216™)	Human	Embryonic kidney	DMEM supplemented with 10% Fetal Calf Serum (FCS), 1% NaPyr, 100 U/mL penicillin and 100 μg/mL streptomycin	37 °C
**hTERT-RPE1** (ATCC^®^ CRL-4000™)	Human	Eye	DMEM:F10 (1:1) supplemented with 10% FCS, 1% NaPyr, 100 U/mL penicillin and 100 μg/mL streptomycin	37 °C
**COS1** (ATCC^®^ CRL-1650™)	Monkey	Kidney	DMEM supplemented with 10% FCS, 1% NaPyr, 100 U/mL penicillin and 100 μg/mL streptomycin	37 °C
**PK1** (ATCC^®^ CRL-101™)	Pig	Kidney	DMEM supplemented with 5% FCS, 1% NaPyr, 100 U/mL penicillin and 100 μg/mL streptomycin	37 °C
**MDCK** (ATCC^®^ CRL-2935™)	Dog	Kidney	DMEM supplemented with 5% FCS, 1% NaPyr, 100 U/mL penicillin and 100 μg/mL streptomycin	37 °C
**CHO** (ATCC^®^ CRL-61™)	Hamster	Ovary	DMEM supplemented with 10% FCS, 1% NaPyr, 100 U/mL penicillin and 100 μg/mL streptomycin	37 °C
**mIMCD-3** (ATCC^®^ CRL-2123™)	Mouse	Kidney	DMEM:F10 (1:1) supplemented with 10% FCS, 1% NaPyr, 100 U/mL penicillin and 100 μg/mL streptomycin	37 °C
**B16-F10** (ATCC^®^ CRL-6475™)	Mouse	Skin	MEM supplemented with 5% FCS, 1% Non-essential amino acid (NEAA), 1% NaPyr, 1.5% MEM vitamins, 100 U/mL penicillin and 100 μg/mL streptomycin	37 °C
**N2A** (ATCC^®^ CCL-131™)	Mouse	Brain	DMEM supplemented with 10% FCS, 1% l-Glutamine, 1% NaPyr, 1% NEAA, 100 U/mL penicillin and 100 μg/mL streptomycin	37 °C
**ATT20** (ATCC^®^ CCL-89™)	Mouse	Pituitary	DMEM supplemented with 7% FCS, 7% HS, 100 U/mL penicillin and 100 μg/mL streptomycin	37 °C
**EL4** (ATCC^®^ TIB-39™)	Mouse	Lymphocyte	Iscove’s medium supplemented with 5% FCS, 100 U/mL penicillin and 100 μg/mL streptomycin	37 °C
**S2** (ATCC^®^ CRL-1963™)	Fly	Embryo	Schneider’s *Drosophila* medium supplemented with 10% heat-inactivated FCS, 50 U/mL penicillin and 50 μg/mL streptomycin	25 °C

ATCC, American Type Culture Collection, Manassas, VA, USA.

## Supplementary Materials

Supplementary materials can be found at http://www.mdpi.com/1422-0067/16/03/5285/s1.
