# Supplementary material for: Species-Dependent Splice Recognition of a Cryptic Exon Resulting from a Recurrent Intronic CEP290 Mutation that Causes Congenital Blindness
Source: Int J Mol Sci. 2015 Mar 9;16(3):5285–98. doi: 10.3390/ijms16035285 (PMC4394476; doi:10.3390/ijms16035285)
Supplement: Supplementary file 1 [file ijms-16-05285-s001.pdf]

Supplementary Information

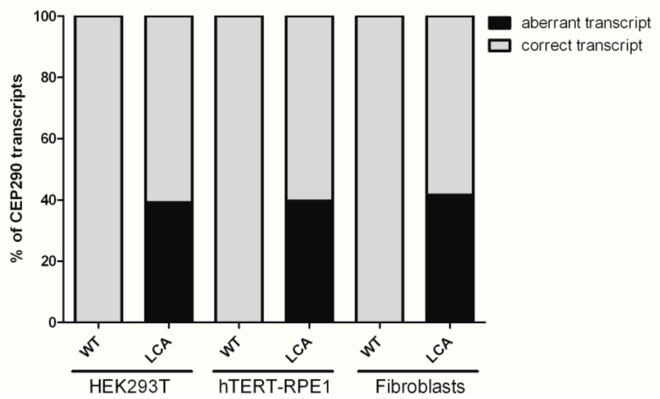

(**A**)

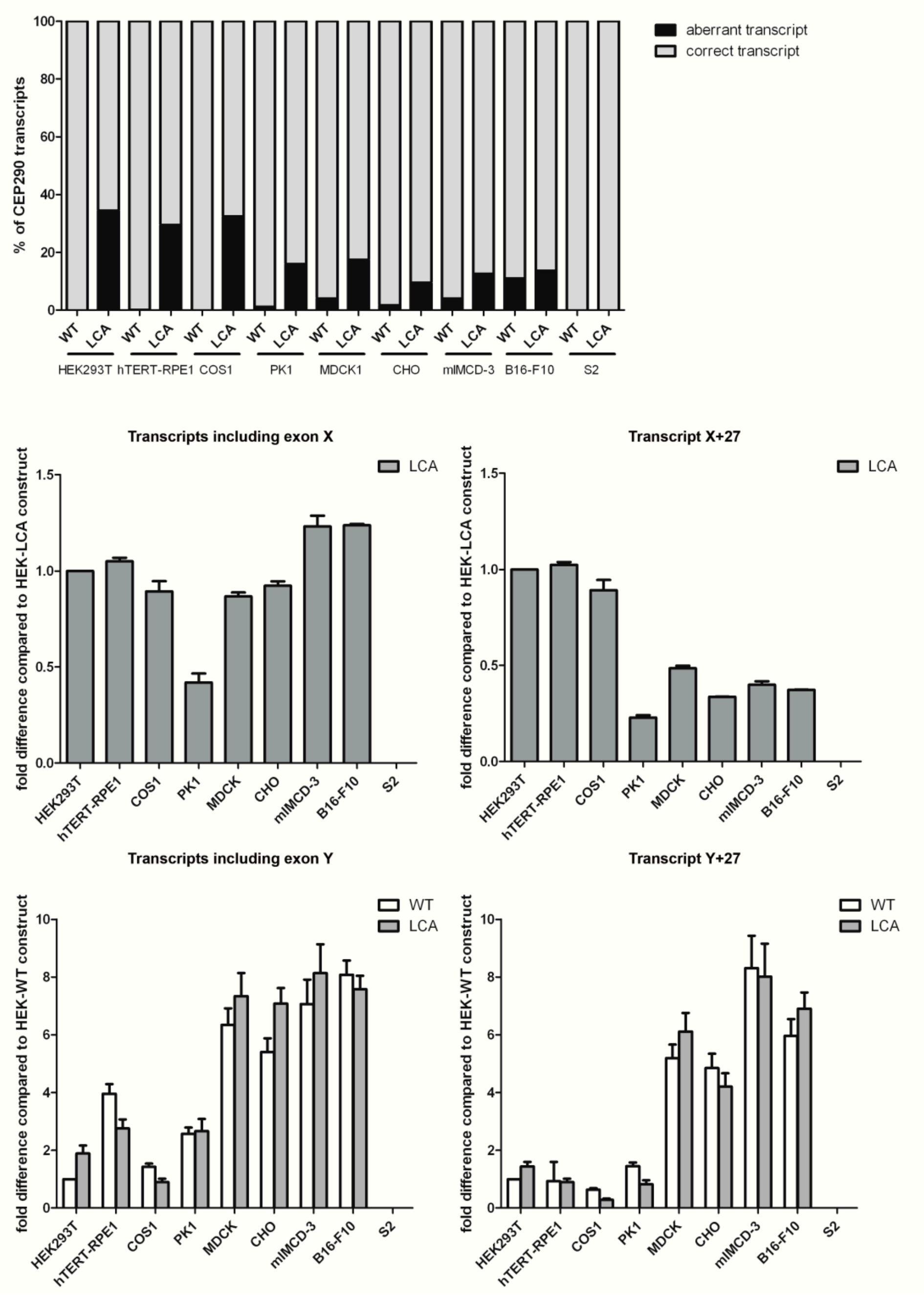

(**B**)

Figure S1. *Cont.*

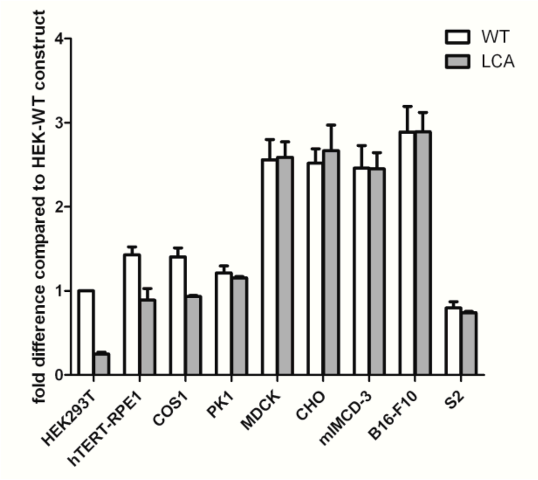

(**B**)

Figure S1. Semiquantitative analysis of the spliced products. (**A**) Densitometric analysis of the gel shown in Figure 1A. Semiquantification of the PCR reaction from exon 26 to exon 27 using the WT and LCA minigene in HEK293T and hTERT-RPE1 cell lines compared to fibroblast cell lines; and (**B**) Semiquantitative analysis of the bands shown in Figure 1B. Upper panel corresponds to the PCR from exon 26 to exon 27 in different cell lines. All exon X-containing transcripts (left panel) and those that include exon X (right panel) as the only cryptic exon in cells transfected with the LCA construct are represented. Exon Y-containing transcripts were also quantified in cell lines transfected with the LCA or WT constructs. Lower panel represents the semiquantitative analysis of exon Z in LCA or WT-transfected cell lines.

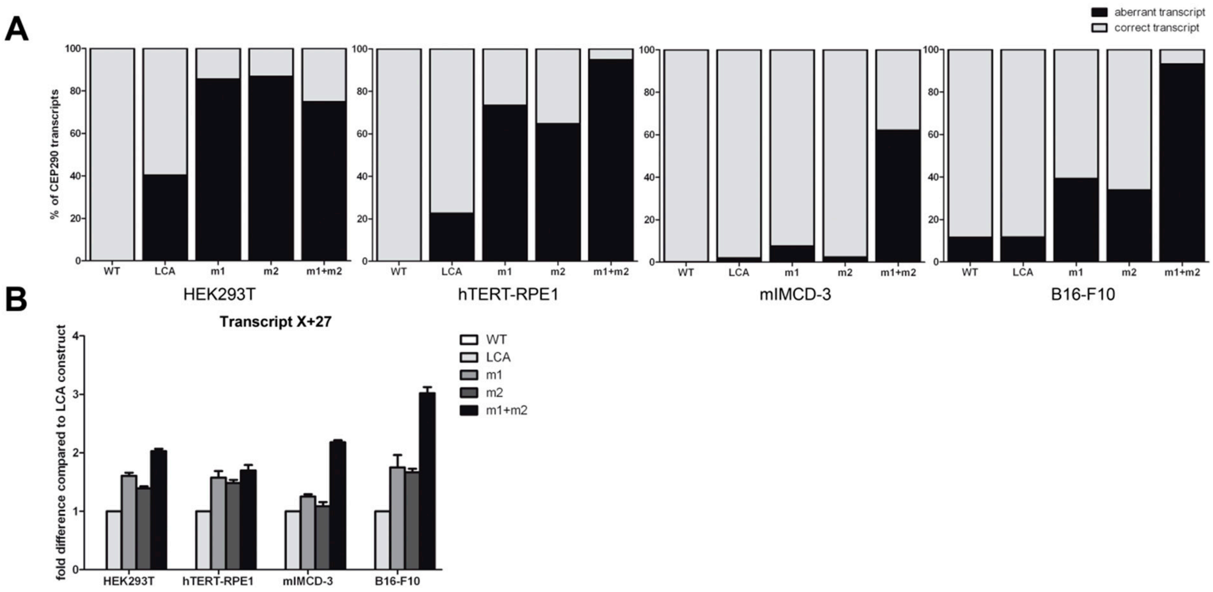

Figure S2. Densitometric analysis of the spliced products in murine and human cell lines. (**A**) Semiquantitative analysis of the PCR reaction from exon 26 to exon 27 depicted in Figure 2C; and (**B**) Semiquantitative analysis of the transcript containing only exon X together with exon 27 in the cell lines transfected with the different constructs. The corresponding gels are shown in Figure 2C; m1 and m2 represent the inserted mutations in the acceptor (m1) or donor (m2) splice sites, or both together (m1 + m2).
